# Tanshinone I promotes angiogenesis and improves ventricular remodeling post-myocardial infarction via ALDH2 signaling-mediated ferroptosis inhibition

**DOI:** 10.3389/fphar.2025.1601017

**Published:** 2025-06-26

**Authors:** Feng Zhang, Lei Li, Qian Liu, Wen-Long Wang, Zhi-Xin Wang, Rong-Qiang Song, Jian Sun, Yi-Yi Zhang, Xin-Chen Ren, Dong Wang, Yu-Ting Wu

**Affiliations:** ^1^ Binzhou Medical University Hospital, Binzhou, China; ^2^ School of Traditional Chinese Medicine, Southern Medical University, Guangzhou, China

**Keywords:** myocardial infarction, ferroptosis, ALDH2, angiogenesis, ventricular remodeling

## Abstract

**Background:**

Tanshinone I (Tan I) has diverse cardioprotective effects, including improving post-myocardial infarction (MI) ventricular remodeling. Ventricular remodeling can be improved by inhibiting endothelial cell (EC) ferroptosis to promote post-MI angiogenesis, but how Tan I does this remains unclear. Thus, we investigated how EC ferroptosis mediates post-MI angiogenesis and Tan I's role in this process.

**Methods:**

*In vivo* experiments: A mouse model of MI was established to evaluate cardiac function, myocardial injury, collagen deposition, and EC ferroptosis. CD31 expression was measured to assess angiogenesis, and western blot analysis was used to detect ALDH2 signaling-related proteins (ALDH2, xCT, GPX4). *In vitro* experiments: Ferroptotic human umbilical vein endothelial cells (HUVECs) induced by erastin were treated with Tan I to assess ferroptosis, cell proliferation, migration, and tubular network formation. Western blot analysis was used to detect ALDH2 signaling-related proteins. Additionally, the role of Tan I was further verified by using the ferroptosis inhibitor ferrostatin-1, the ALDH2 agonist Alda-1, or the ALDH2 inhibitor daidzein *in vitro* and *in vivo* models, respectively.

**Results:**

We found that Tan I improved post-infarction cardiac function and myocardial injury, inhibited post-infarction collagen deposition and EC ferroptosis, and promoted CD31 expression *in vivo* by activating ALDH2 and promoting ALDH2 signaling-related protein levels. Additionally, Tan I inhibited ferroptosis, promoted proliferation and migration, and enhanced tubular network formation in HUVECs *in vitro* by activating ALDH2 and promoting ALDH2 signaling-related protein levels.

**Conclusion:**

Tan I may improve ventricular remodeling by activating ALDH2 signaling, inhibiting EC ferroptosis, and promoting angiogenesis.

## Graphical Abstract

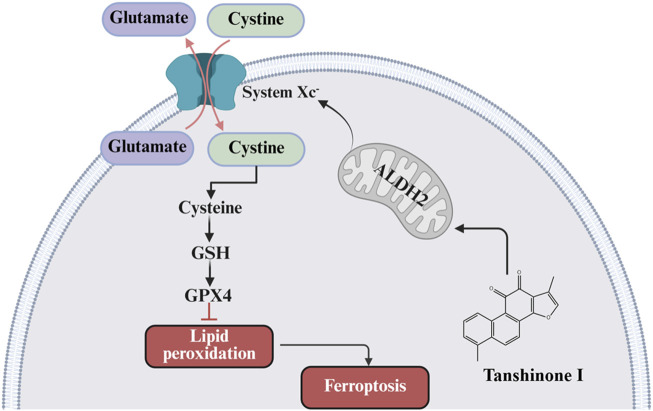

## 1 Introduction

Myocardial infarction (MI) represents a major factor inducing mortality globally (Saglietto et al., 2021), and alterations of ventricular morphology, structure, size and function post-MI are major determinants of the incidence of post-infarction cardiac events (e.g., heart failure, HF) and long-time prognostic outcome (Zhao et al., 2019; Riddell et al., 2020). Therefore, the better prevention and control of post-infarction ventricular remodeling remains a prominent challenge in current cardiovascular disease (CVD) research.

The post-MI myocardial repair and prognostic outcome are tightly associated with the functional status of vascular endothelial cells (ECs), while rapid angiogenesis in the infarcted area and residual blood vascular reopening are essential for establishing a good collateral circulation to improve blood supply and myocardial survival in the infarcted area (Zhang B. et al., 2022). Additionally, therapeutic neovascularization increases blood vessel numbers in the infarct border zone, which better perfuses the endangered myocardial tissue, delays MI progression, protects cardiac function and improves ventricular remodeling (Wu X. et al., 2021). Nonetheless, its exact mechanism is unknown.

Ferroptosis, an iron-dependent form of regulated cell death distinct from apoptosis, is driven by elevated lipid reactive oxygen species (ROS), lipid peroxidation, and dysregulated iron metabolism (Li et al., 2022). Cardiomyocyte ferroptosis contributes to the onset and progression of cardiovascular diseases (CVDs), including post-infarction ventricular remodeling and HF. Notably, ferroptosis inhibitors or iron chelators ameliorate cardiac dysfunction and protect against ischemic myocardial injury (Fang et al., 2019; Wu X. et al., 2024). Consistent with this, we previously demonstrated that the ferroptosis inhibitor ferrostatin-1 (Fer-1) significantly reduces collagen deposition and attenuates ventricular remodeling post-MI (Wu et al., 2023). Moreover, ferroptosis in vascular endothelial cell (EC) disrupts angiogenesis, a critical reparative process post-MI, whereas ferroptosis inhibitors or iron chelators enhance neovascularization (Yuan et al., 2022). Given that impaired angiogenesis exacerbates cardiac repair and adverse remodeling, targeting ferroptosis in post-MI ECs may represent a therapeutic strategy to promote angiogenesis and thereby improve ventricular remodeling outcomes.

Acetaldehyde dehydrogenase 2 (ALDH2), a mitochondrial enzyme critical for detoxifying reactive aldehydes (e.g., 4-HNE and malondialdehyde) generated during oxidative stress, is highly expressed in the heart, brain, and lungs (Gomes et al., 2015; Zhang et al., 2018). ALDH2’s cardioprotective effects are well-established, and its activation or overexpression attenuates post-MI injury by reducing toxic aldehyde accumulation, mitigating oxidative stress-induced cardiomyocyte apoptosis, and improving infarct healing, thereby preserving cardiac function and inhibiting ventricular remodeling. On the contrary, ALDH2 inhibition or knockdown exacerbates the endoplasmic reticulum stress-induced cardiac dysfunction and increases the susceptibility to myocardial injury (Guo et al., 2022; Lai et al., 2022). Beyond cardiomyocytes, ALDH2 activation improves vascular endothelial function, protects against vascular endothelial injury, and promotes angiogenesis (Li et al., 2015; Hua et al., 2018; Sidramagowda Patil et al., 2020). Notably, emerging evidence suggests ALDH2 may regulate ferroptosis, a process tightly linked to oxidative stress, via glutathione peroxidase 4 (GPX4) stabilization (Zhang et al., 2025). However, whether ALDH2-dependent ferroptosis inhibition in ECs directly drives angiogenesis to ameliorate post-MI remodeling remains unexplored.

As an effective compound in Salvia miltiorrhiza, tanshinone I (Tan I) exerts favorable pharmacokinetic profiles and established safety in traditional medicine (Ke et al., 2023), and exhibits diverse cardiovascular protective effects against inflammation, apoptosis, oxidation, and cardiomyocyte ferroptosis (Wu et al., 2019; Wu Y. T. et al., 2021). However, whether Tan I inhibits vascular EC ferroptosis, promotes angiogenesis, and inhibits ventricular remodeling post-MI through regulating ALDH2 signaling remains to be further determined. Therefore, we constructed a ferroptosis model *in vitro* using erastin-induced human umbilical venous ECs (HUVECs) and a post-MI ventricular remodeling mouse model through ligating the left anterior descending (LAD) artery, so as to investigate the potential mechanism by which ALDH2 signaling influenced the functions of Tan I in vascular EC ferroptosis, angiogenesis, as well as ventricular remodeling post-MI. The present study provides a basis for identifying effective drugs and therapeutic targets that may prevent ventricular remodeling post-MI in clinical practice.

## 2 Materials and methods

### 2.1 Materials

Antibodies against ALDH2 (15310-1-AP), glutathione peroxidase 4 (GPX4, ab125066), recombinant solute carrier family 7, member 11 (xCT, ab175186), and GAPDH (AF7021) were provided by Proteintech (Chicago, IL, United States), Abcam (Cambridge, UK), and Affinity Biosciences (Cincinnati, OH, United States), respectively. Meanwhile, ferrous ions, malondialdehyde (MDA), superoxide dismutase (SOD), reduced glutathione (GSH), and mitochondrial ALDH2 assay kits were provided by Solarbio (Beijing, China). An ROS detection kit was acquired from Beyotime Biotechnology (Shanghai, China). Erastin, Ferrostatin-1 (Fer-1), Alda-1, and daidzein were acquired from MedChem Express (Monmouth Junction, NJ, United States). Tan I (purity ≥98%) was provided by Chengdu Manstar Biotechnology Co. (Chengdu, China). Matrigel was purchased from Corning (Corning, NY, United States). All the other major reagents were purchased from BioReagents Inc.

### 2.2 HUVEC culture

HUVECs provided by Zhongqiao Xinzhou Biotechnology Co. (Shanghai, China) were cultivated within EC Basal Medium that contained EC Medium Additives (Zhongqiao Xinzhou) at 37°C with 5% CO_2_. For all the experiments, cells from the first 5 passages were used.

### 2.3 Preparation and grouping of MI animal models

Six-week-old C57BL/6 male mice (weight about 22 g, Pengyue Laboratory Animal Breeding Co., Ltd., Jinan, China) were reared within the specific-pathogen-free animal room of Binzhou Medical University Hospital (Binzhou, China). Every experiment was performed strictly following ethical guidelines released by the Animal Ethics Committee of the Binzhou Medical University Hospital (ethical review number: 20231208-17). Referring to the modeling method used by our research group (Chen et al., 2022; Wu Y. T. et al., 2024), the MI animal model was constructed via ligating LAD artery. First, animals were administered 1% pentobarbital sodium at 0.1 mL/10 g for anesthesia, and then fixed on an anatomical table in the supine position. The skin on the chest was cut open along the left fourth and fifth intercostal spaces, followed by layer-by-layer blunt separation of the subcutaneous tissue for fully exposing intercostal spaces. Further, ophthalmic scissors were used to cut the intercostal tissues along the intercostal spaces. After the heart was completely exposed using a chest dilator, the LAD artery was ligated under a microscope. When electrocardiography revealed ST-segment elevation accompanied by whitening between ligation site and cardiac apex, it indicated the successful model construction. The mice were then randomly divided into ferroptosis inhibitor (ferrostatin-1, Fer-1, 5 mg/kg), ALDH2 agonist (Alda-1, 10 mg/kg), ALDH2 inhibitor (daidzein, 100 mg/kg), high-dose Tan I (10 mg/kg), low-dose Tan I (5 mg/kg), and captopril (20 mg/kg) groups, six in each group. Among these groups, pharmacological interventions were initiated at 1 day post-MI induction at the same time daily. Specifically, Fer-1, Alda-1, daidzein, and captopril were administered via intraperitoneal injection, while high- and low-dose Tan I were delivered through oral gavage. The sham operated group underwent identical thoracotomy procedures without coronary artery ligation and received equivalent volumes of normal saline via daily gavage. At 28 days post-MI, comprehensive cardiac functional assessment was performed using cardiac ultrasonography, followed by tissue collection for subsequent histopathological analysis and related indicator testing.

### 2.4 Cardiac ultrasonography

Cardiac ultrasonography was performed 28 days later. After the chest skin was totally exposed, mice were anesthetized using a gas instrument and fixed on a table. The heart structure and function were then measured using a high-resolution small-animal ultrasound imaging system (VisualSonics, Toronto, Canada). To assess the mouse heart function, both left ventricular end-diastolic and end-systolic diameters (LVIDd and LVIDs) were examined, whereas left ventricular ejection fraction (LVEF) and fractional shortening (LVFS) were computed based on M-mode images through software analyses. To ensure the accuracy of the results, all procedures were performed by professional cardiac ultrasound physicians.

### 2.5 Histopathology testing

Paraffin-embedded heart tissues were sectioned at 5-μm thickness using a Leica RM2235 microtome, mounted on poly-L-lysine-coated slides, and dried at 37°C overnight. Sections were deparaffinized in fresh xylene (2 × 10 min), rehydrated through graded ethanol (100% × 2, 95%, 85%, 70%; 5 min each), and rinsed in distilled water. HE staining was performed using hematoxylin staining solution (C0107-100mL, Beyotime Biotechnology, Shanghai, China) for 5–8 min, followed by differentiation in 1% acid alcohol (5–10 s), bluing in tap water (10 min), and counter staining with eosin staining solution (C0105S, Beyotime Biotechnology, Shanghai, China) for 30 sec-2 min. Masson staining was performed using a commercial kit (D026-1-1, Nanjing Jiancheng Bioengineering Institute), and all procedures were carried out according to the manufacturer’s instructions. All sections were dehydrated in ethanol, cleared in xylene, and mounted with Neutral Balsam (C0173, Beyotime Biotechnology, Shanghai, China). Images were acquired using a pathological slide scanner, and collagen volume fraction in Masson-stained sections was analyzed via ImageJ software.

### 2.6 Ferrous iron, ROS, MDA, GSH, SOD, and ALDH2 activity measurements

Ferrous iron, ROS, MDA, GSH, SOD levels, and ALDH2 activity were quantified using commercial assay kits according to manufacturers' protocols. ROS levels were determined using a Cytation5 cell imaging multimode microplate reader (Agilent Technologies, Santa Clara, CA, United States) at excitation/emission wavelengths of 480/525 nm. Ferrous iron, MDA, GSH, and SOD levels, as well as ALDH2 activity, were measured at 593, 532, 405, 560, and 340 nm, respectively, using an automated microplate reader. All assays were performed in triplicate, and data were normalized to protein concentration (BCA assay, P0012, Beyotime Biotechnology, Shanghai, China).

### 2.7 Molecular docking and molecular dynamics analyses

The crystal structure of ALDH2 (PDB ID: 8DR9) was retrieved from the RCSB Protein Data Bank (http://www.rcsb.org/pdb), and the three-dimensional structure of Tan I (ZINC:2558154) was downloaded from the ZINC database (https://zinc.docking.org). Pre-docking preparation: The ALDH2 structure was processed using AutoDockTools-1.5.7 to remove water molecules, add polar hydrogens, and assign Kollman charges. Tan I was subjected to energy minimization using the MMFF94 force field in Open Babel 3.1.1. Molecular docking was performed using AutoDock Vina 1.2.3 with a grid box centered on the active site. The exhaustiveness parameter was set to 20, and the top 10 binding poses were retained. Molecular dynamics (MD) simulations were carried out using YASARA Structure 22.1.24 with the AMBER14 force field. The ALDH2-Tan I complex was solvated in a cubic water box with a 10 Å padding, neutralized with NaCl, and equilibrated at 300 K for 100 ps under periodic boundary conditions. Production MD runs were performed for 100 ns with a 2 fs time step, saving coordinates every 100 ps. Trajectory analysis was performed using built-in YASARA tools to calculate the root-mean-square deviation (RMSD), and binding free energy via the MM/PBSA method. All simulations were repeated three times to ensure reproducibility, and software parameters were logged using a custom Python script to maintain consistency across experimental batches.

### 2.8 Surface plasmon resonance (SPR) detection

The interaction between Tan I and ALDH2 was investigated using a PlexArray HT A100 SPR instrument (Plexera LLC, Woodinville, WA, United States) for real-time monitoring. Briefly, an ALDH2-immobilized 3D dextran chip was placed into the flow chamber. Subsequently, Tan I samples at various concentrations (0.625, 1.25, 2.5, 5, and 10 µM) were introduced for assessment. Phosphate-buffered saline (PBS, pH 7.4) served as the running buffer throughout the experiment, while a 10 mM glycine–HCl buffer (pH 2.0) was utilized for chip regeneration after each measurement cycle. All experimental procedures adhered strictly to the manufacturer-provided protocols. Data analysis was conducted with BIA Evaluation Version 3.0 Software (Biacore AB Corporation, Uppsala, Sweden). This software was employed to generate kinetic curves and compute kinetic parameters, including binding affinity between Tan I and ALDH2, to characterize their interaction.

### 2.9 Angiogenesis assay

Tube formation assays were performed on control HUVECs and cells treated with erastin (10 µM), erastin combined with Tanshinone I (2.5, 5, 10 µM), Alda-1 (10 µM), daidzein (10 µM), or Fer-1 (10 µM). Matrigel (Corning, 356234, LDEV-free, NY, United States) was thawed overnight at 4°C, diluted 1:1 with EC Basal Medium, and pipetted into 24-well plates (500 μL/well). Plates were incubated at 37°C for 1 h to allow polymerization. HUVECs were centrifuged, resuspended in EC Basal Medium, and seeded onto Matrigel-coated wells at 1 × 10^5^ cells/well in triplicate. Following incubation at 37°C, 5% CO_2_ for 6 h, tubular network formation was visualized under a phase-contrast microscope.

### 2.10 Cell proliferation assay

After harvesting, digestion and centrifugation of logarithmic-phase HUVECs, cells (about 5 × 10^3^ cells/well) were seeded in 96-well plates for different groups, including control, erastin (10 µM), Tan I (2.5, 5, 10 µM), Alda-1 (10 µM), daidzein (10 µM), and Fer-1 (10 µM). Subsequently, a CCK-8 assay kit (Solarbio) was used to measure cell proliferation capacity.

### 2.11 Wound healing assay

The logarithmic-phase HUVECs (5 × 10^5^ cells/well) were seeded in 6-well plates and subjected to corresponding treatments for 24 h. A 10-µL pipette tip was then used to create two horizontal scratches along the bottom of the wells. Detached cells were washed with phosphate-buffered saline (PBS), and the medium was replaced with EC medium containing 1% fetal bovine serum (FBS) for further incubation. An inverted microscope was used to observe the changes in relative migration and capture images at 0 and 24 h.

### 2.12 Transwell assay

The logarithmic-phase HUVECs received erastin (10 µM) and erastin combined with Tan I (2.5, 5, 10 µM), Alda-1 (10 µM), daidzein (10 µM) or Fer-1 (10 µM) treatments. Subsequently, 0.5 mL cell suspension from each group was introduced into top chamber, whereas 10% FBS-containing EC medium was added into bottom chamber to incubate for 12 h with 5% CO_2_ under 37°C. After discarding the medium, the chamber was rinsed thrice by PBS, followed by additional 20 min of fixation using 4% paraformaldehyde. Following washing thrice by PBS again, cells underwent 15 min of crystal violet staining. Subsequently, the chamber was cleaned with PBS, while wet cotton swabs were utilized to wipe non-migrating cells from the top chamber. Five fields were randomly selected, and cells were counted using the inverted microscope.

### 2.13 CD31 immunohistochemical staining

The heart tissue sections were dewaxed, rehydrated, antigen-retrieved, blocked with a 5% goat serum blocking solution for 2 h under ambient temperature, and then rinsed by PBS thrice. Subsequently, the CD31 primary antibody (1:100) was added for overnight section incubation under 4°C, followed by additional 2 h of secondary antibody incubation under ambient temperature after PBS washing thrice. Sections were then washed by PBS thrice again, prior to diaminobenzidine (DAB) coloration and hematoxylin re-staining. Finally, these sections were sealed under a microscope to observe CD31 expression.

### 2.14 Western blotting assay

HUVECs and myocardial tissue protein samples were prepared for different groups. In brief, HUVECs and tissues were subjected to lysis with the lysis solution (including protease/phosphatase inhibitors; Beyotime), and later protein supernatants were collected via centrifugation. After quantitative denaturation, we loaded 15 µg cellular or 60 µg cardiac tissue protein samples onto the 10% sodium dodecyl sulfate (SDS) protein gel for electrophoresis before transferring them on the polyvinylidene fluoride (PVDF) membrane that was closed using 5% bovine serum albumin (BSA) sealing solution for a 1.5-h duration under ambient temperature. Target proteins then underwent overnight primary antibody incubation against ALDH2 (Rabbit, 1:2500), xCT (Rabbit, 1:10000), GPX4 (Rabbit, 1:10000), and GAPDH (Rabbit, 1:1000) under 4°C on a shaker. After Tris-buffered saline Tween (TBST) washing thrice, sections received additional 1 h of secondary antibody incubation under ambient temperature on a shaker. Subsequently, cells were rinsed thrice by TBST, while an ECL light solution (Boster Bio, Pleasanton, CA, United States) was adopted for developing target protein. ImageJ was employed for result analysis.

### 2.15 Statistical analysis

All data were presented as the mean ± standard deviation. Statistical analyses were performed using IBM SPSS Statistics, Version 28.0 (IBM Corp., Armonk, NY, United States). One-way analysis of variance (ANOVA) was used to analyze between-group differences. Prior to ANOVA, Levene’s test for homogeneity of variances was conducted. If variances were homogeneous, Bonferroni *post hoc* tests were performed for pairwise comparisons. If variances were heterogeneous, Welch’s ANOVA with Dunnett’s T3 tests was used to assess overall and pairwise differences. *P < 0.05* stood for statistical significance. All experiments were independently repeated at least three times.

## 3 Results

### 3.1 Tan I inhibits EC ferroptosis and promotes angiogenesis to improve post-MI ventricular remodeling

**FIGURE 1:**
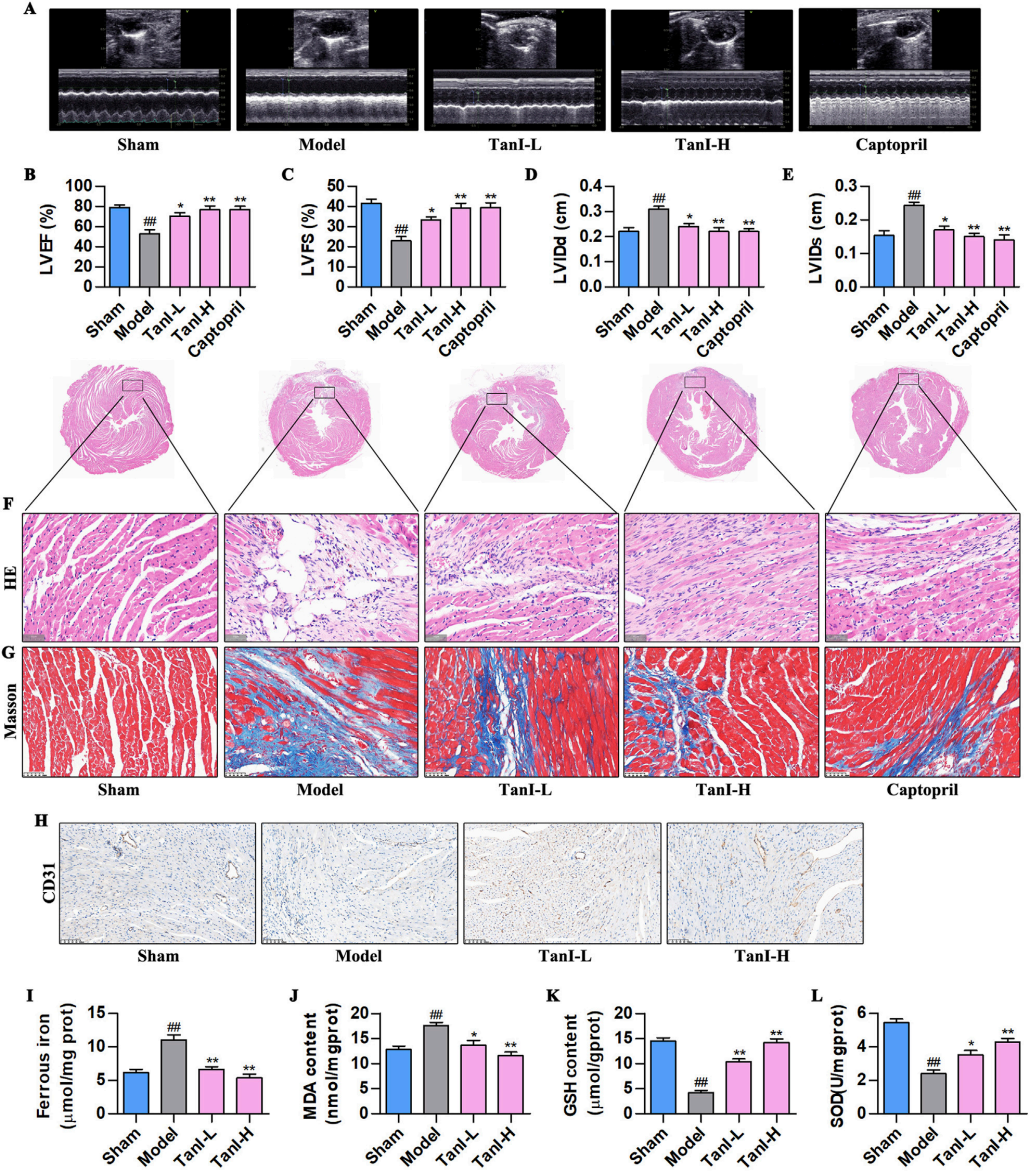
Tan I inhibits EC ferroptosis and promotes angiogenesis to improve ventricular remodeling post-MI. **(A)** Transthoracic echocardiography test. **(B–E)** LVEF, LVFS, LVIDd, and LVIDs levels (n = 3, *P < 0.01*). **(F)** HE staining. **(G)** Masson staining. **(H)** CD31 protein level was detected using immunohistochemistry. **(I–L)** Ferrous iron, MDA, GSH, and SOD contents (n = 3, *P < 0.01*). ^*#*^ *P < 0.05*, ^*##*^ *P < 0.01* vs. sham group. *P < 0.05, **P < 0.01 vs. model group.

Transthoracic echocardiography was performed for observing functions of Tan I in post-MI cardiac function. As a result, LVEF and LVFS dramatically declined, whereas LVIDd and LVIDs were markedly elevated in the model group relative to the sham group (Figures 1A–E, *P < 0.01*). Tan I and captopril apparently elevated LVEF and LVFS but declined LVIDd and LVIDs compared with the model group (Figures 1A–E, *P < 0.01*), improving post-infarction cardiac function. Further, HE and Masson staining were performed for observing functions of Tan I in post-infarction myocardial injury. Typically, HE staining revealed excessive infiltration of necrotic cardiomyocytes and inflammatory cells, disturbed arrangement of cardiomyocytes, disappearance of myocardial transverse striations, and overgrowth of fibrous tissues in the model group, whereas Tan I and captopril significantly improved post-infarction myocardial injury (Figure 1F). Masson staining (Figure 1G) showed the massive collagen deposition in the model group, whereas Tan I and captopril inhibited post-infarction collagen deposition. These results suggest that Tan I improves post-infarction cardiac function and inhibits post-infarction ventricular remodeling.

In addition, promoting angiogenesis following MI can improve post-infarction ventricular remodeling (Guo et al., 2020). This work used immunohistochemistry for detecting CD31 expression, a marker of angiogenesis, and found that CD31 was weakly expressed in the model group, while Tan I promoted its expression dose-dependently (Figure 1H). EC ferroptosis has a key effect on regulating angiogenesis (Xia et al., 2022). For investigating whether Tan I’s function after MI was related to ferroptosis inhibition, ferroptosis-related indicators were detected. As shown in Figures 1I–L, the ferrous iron and MDA contents apparently elevated, and antioxidant products GSH and SOD dramatically decreased within myocardial tissues in the model group following MI relative to the sham group. However, Tan I decreased ferrous iron and MDA contents, whereas increased GSH and SOD levels dose-dependently (*P < 0.01*). Taken together, Tan I may promote angiogenesis and improve ventricular remodeling post-MI by inhibiting EC ferroptosis.

### 3.2 Tan I activates ALDH2 and promotes ALDH2 signaling-related protein levels

**FIGURE 2:**
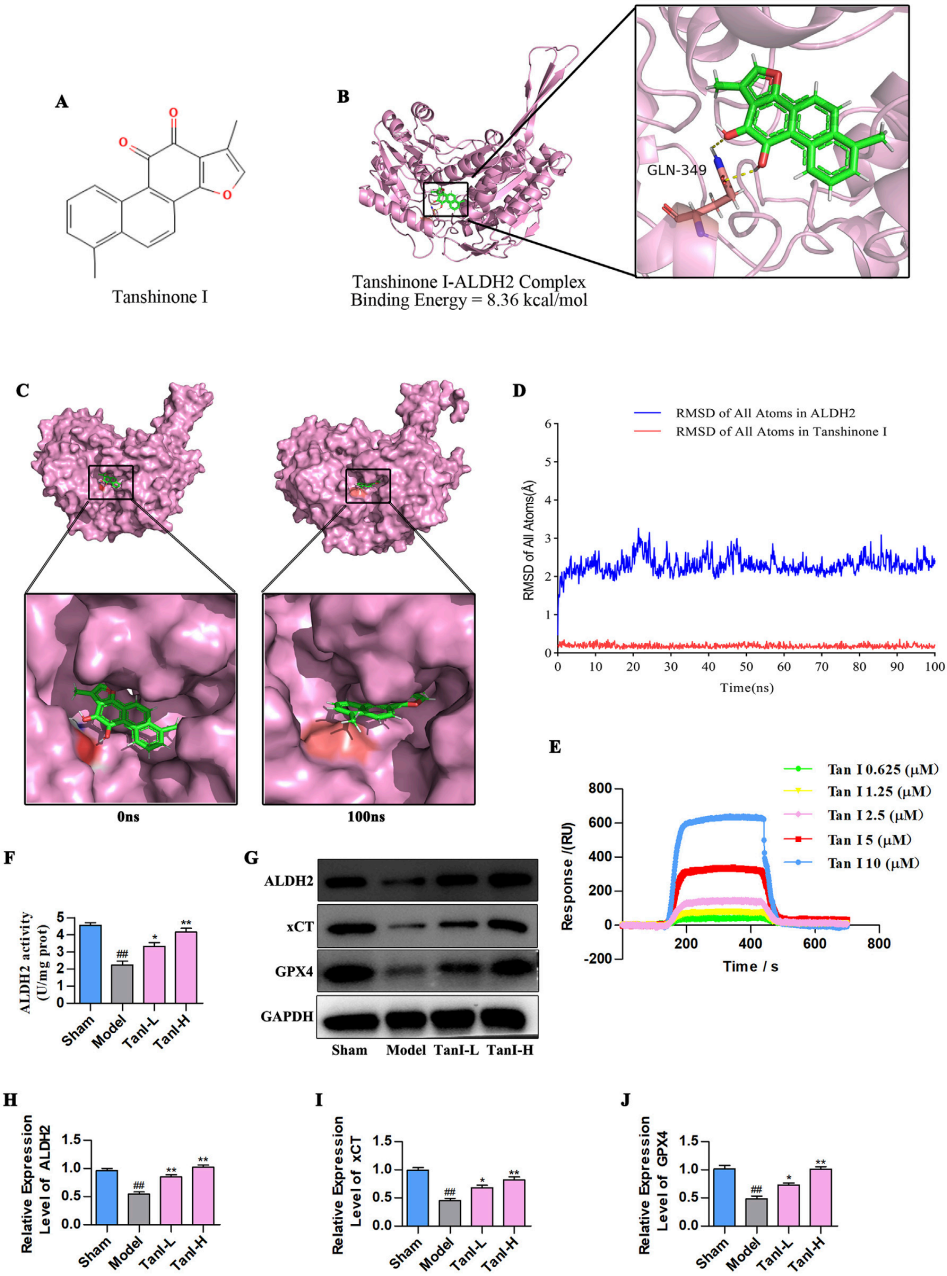
Tan I activates ALDH2 and promotes ALDH2 signaling-related protein levels. **(A)** Structure of the tan I compound. **(B)** The Tan I 3D crystal structure (ZINC:2558154) within the complex with ALDH2 (PDB ID:8DR9). Tan I is denoted as green, whereas hydrogen bonds as yellow lines. **(C)** Surface presentation for the Tan I–ALDH2 complex crystal structure at 0 and 100 ns. **(D)** RMSD of all atoms. **(E)** SPR binding curves of Tan I at varying concentrations to ALDH2. **(F)** ALDH2 activity (*n = 3, P < 0.01*). **(G–J)** ALDH2, xCT, and GPX4 protein levels were measured through western blotting (n = 3, *P < 0.01*). ^*#*^ *P < 0.05*, ^*##*^ *P < 0.01* vs. sham group. ^*^ *P < 0.05*, **P < 0.01 vs. model group.

As revealed by molecular docking study, Tan I (Figure 2A) directly targeted ALDH2, and the Tan I–ALDH2 complex had a binding energy of −8.36 kcal/mol, indicating good binding affinity. In addition, the 3D crystal structure of the complex suggested that Tan I generated hydrogen bonds with the GLN-349 amino acid residues of ALDH2 (Figure 2B). According to the molecular dynamics results (Figure 2C), the crystal structure of the complex was stable from 0 ns to 100 ns at the binding site in the center of the complex. With regard to heavy atoms in this complex, their root mean square deviation (RMSD) was maintained at 2.2 Å during the evolution of the blue line RMSD of all atoms in ALDH2 from 0 ns to 100 ns. Meanwhile, the red line RMSD of all atoms in Tan I was maintained in the pocket combined with ALDH2 (Figure 2D). Furthermore, we employed SPR to detect the interaction between TanI and ALDH2. The binding affinity between TanI and ALDH2 was 1.91 × 10^−8^ M, indicating that TanI could directly bind to ALDH2 (Figure 2E). To further validate this interaction, the ALDH2 activity and expression were detected using ALDH2 activity detect kit and western blotting, respectively. As a result, its activity and expression evidently declined in the model group versus the sham group, while Tan I enhanced ALDH2 activity and promoted ALDH2 expression dose-dependently (Figures 2F–H, *P < 0.01*). Additionally, the xCT and GPX4 expression in the model group was remarkably lower than the sham group, while Tan I dose-dependently promoted their expression (Figures 2G,I,J, P *< 0.01*). In line with these findings, Tan I may inhibit EC ferroptosis and promote angiogenesis post-MI by activating ALDH2 signaling.

### 3.3 Fer-1 suppresses EC ferroptosis and enhances angiogenesis post-MI via activating ALDH2 signaling

**FIGURE 3:**
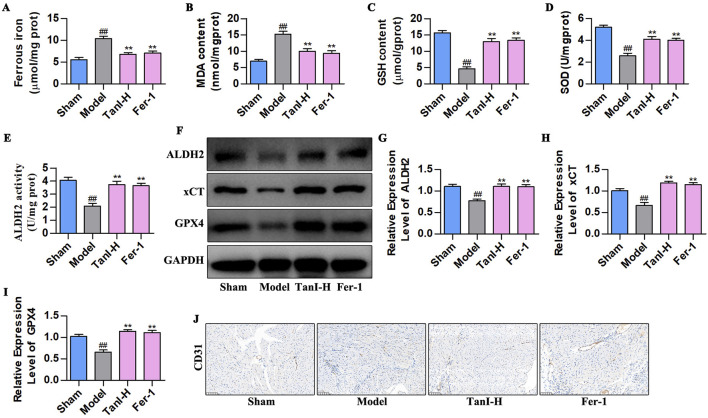
Fer-1 suppresses EC ferroptosis and enhances angiogenesis post-MI via activating ALDH 2 signaling. **(A–D)** Ferrous iron, MDA, GSH, and SOD contents (n = 3, *P < 0.01*). **(E)** ALDH2 activity (n = 3, *P < 0.01*). **(F–I)** ALDH2, xCT and GPX4 protein levels were measured using western blotting (n = 3, *P < 0.01*). **(J)** CD31 protein level was detected via immunohistochemistry. ^*#*^ *P < 0.05*, ^*##*^ *P < 0.01* vs. sham group. *P < 0.05, **P < 0.01 vs. model group.

Considering that Fer-1 inhibits ferroptosis in ECs (Lv et al., 2023), we used Fer-1 to observe the effects and regulatory mechanisms of Tan I in EC ferroptosis post-MI. Figures 3A–D display the indicators related to ferroptosis. As a result, ferrous iron and MDA contents remarkably increased in the model group, whereas GSH and SOD contents evidently declined (*P < 0.01*) relative to the sham group, whereas Fer-1 lowered ferrous iron and MDA contents and elevated GSH and SOD levels (*P < 0.01*), consistent with results obtained for Tan I. Additionally, further detection of ALDH2 activity and ALDH2 signaling-related proteins (ALDH2, xCT, and GPX4) revealed that ALDH2 activity and ALDH2, xCT, and GPX4 protein levels markedly declined in the model group relative to the sham group, whereas Fer-1 promoted their levels, conforming to the findings obtained for Tan I (Figures 3E–I, *P < 0.01*). Additionally, CD31 expression in the model group dramatically decreased relative to the sham group, whereas Fer-1 promoted its expression, consistent with the Tan I results (Figure 3J). Collectively, Tan I suppresses EC ferroptosis and promotes angiogenesis post-MI by activating ALDH2 signaling.

### 3.4 Inhibiting ALDH2 enhances EC ferroptosis and suppresses angiogenesis post-MI

**FIGURE 4:**
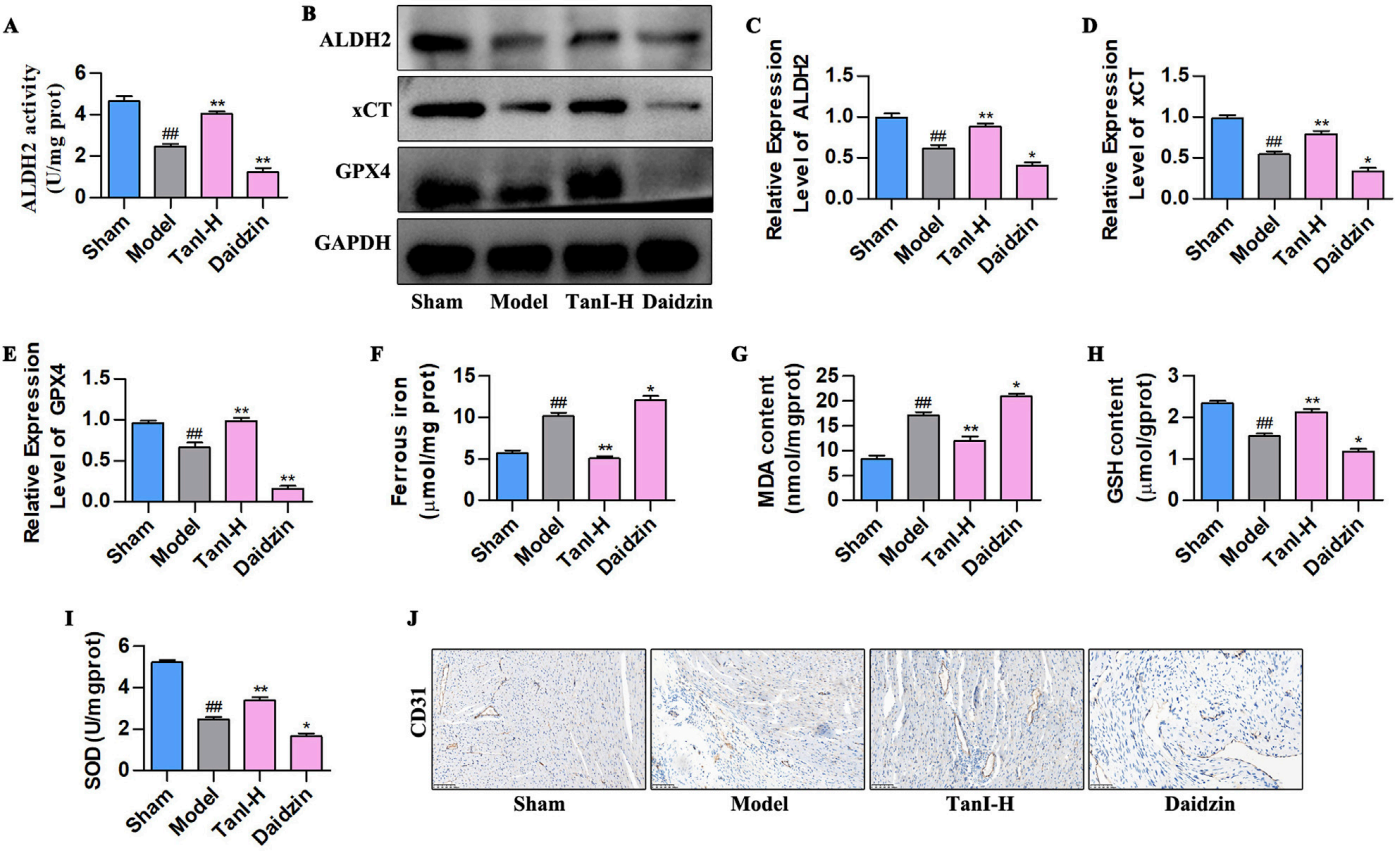
ALDH2 inhibition enhances EC ferroptosis and suppresses angiogenesis post-MI. **(A)** ALDH2 activity (n = 3, *P < 0.01*). **(B–E)** ALDH2, xCT, and GPX4 protein levels were measured using western blotting (n = 3, *P < 0.01*). **(F–I)** Ferrous iron, MDA, GSH, and SOD contents (n = 3, *P < 0.01*). **(J)** CD31 protein level was detected via immunohistochemistry. ^*#*^ *P < 0.05*, ^*##*^ *P < 0.01* vs. sham group. *P < 0.05, **P < 0.01 vs. model group.

Daidzein (the selective ALDH2 inhibitor) was adopted for verifying how ALDH2 signaling affected Tan I - mediated suppression of EC ferroptosis and promotion of angiogenesis post-MI. According to the ALDH2 activity analysis results, daidzein suppressed ALDH2 activity (Figure 4A, *P < 0.01*), contrary to results of Tan I, which enhanced ALDH2 activity (Figure 4A, *P < 0.01*). Besides, ALDH2 signaling-related protein levels were detected through western blotting, which showed that daidzein inhibited ALDH2, xCT, and GPX4 levels (Figures 4B–E, *P < 0.01*), different from the results of Tan I that promoted ALDH2 signaling (Figures 4B–E, *P < 0.01*). Ferroptosis-related indicators were further detected, as a result, daidzein increased ferrous iron and MDA contents, whereas decreased GSH and SOD contents relative to the model group (Figures 4F–I, *P < 0.01*), different from findings obtained for Tan I which decreased ferrous iron and MDA contents and increased GSH and SOD contents (Figures 4F–I, *P < 0.01*). Further analysis revealed that daidzein inhibited CD31 expression, whereas Tan I promoted its expression (Figure 4J). Taken together, these results demonstrate that ALDH2 inhibition enhances EC ferroptosis and suppresses angiogenesis post-MI.

### 3.5 Promoting ALDH2 signaling inhibits EC ferroptosis to enhance angiogenesis post-MI

**FIGURE 5:**
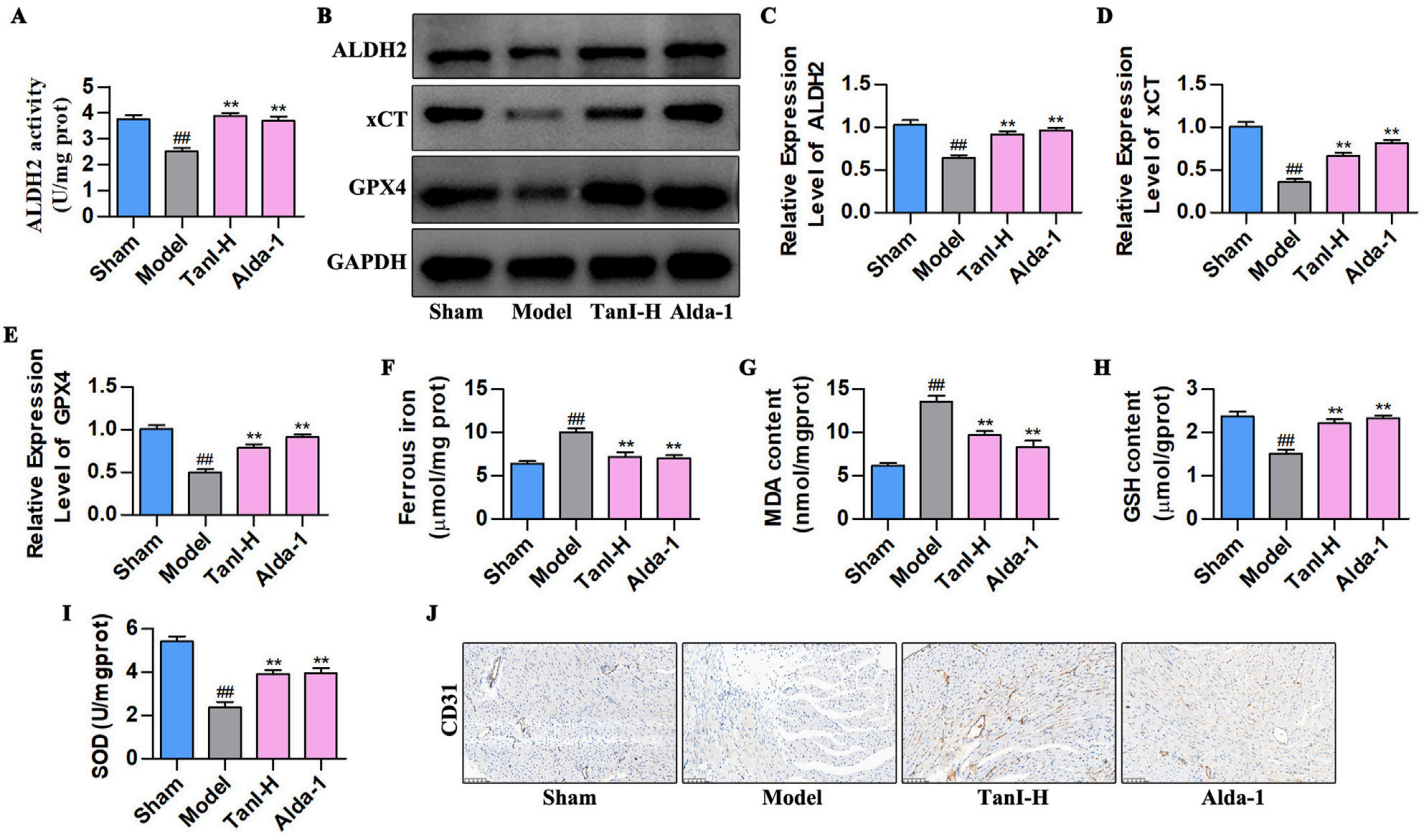
Promoting ALDH2 signaling inhibits EC ferroptosis to enhance angiogenesis post-MI. **(A)** ALDH2 activity (n = 3, *P < 0.01*). **(B–E)** ALDH2, xCT, and GPX4 protein levels were measured using western blotting (n = 3, *P < 0.01*). **(F–I)** Ferrous iron, MDA, GSH, and SOD contents (n = 3, *P < 0.01*). **(J)** CD31 protein level was detected via immunohistochemistry. ^*#*^ *P < 0.05*, ^*##*^ *P < 0.01* vs. sham group. *P < 0.05, **P < 0.01 vs. model group.

Alda-1 (the selective ALDH2 agonist) was adopted for validating how ALDH2 signaling affected the Tan I - mediated inhibition of EC ferroptosis and promotion of angiogenesis post-MI. As revealed by the ALDH2 activity analysis results, Alda-1 enhanced ALDH2 activity (Figure 5A, *P < 0.01*), conforming to effects of Tan I which enhanced ALDH2 activity (Figure 5A, *P < 0.01*). ALDH2, xCT, and GPX4 levels were detected by western blotting, as a result, Alda-1 promoted their levels in comparison with the model group (Figures 5B–E, *P < 0.01*), consistent with the effects of Tan I that promoted ALDH2 signaling (Figures 5B–E, *P < 0.01*). Moreover, ferroptosis-related indicators were detected, revealing that Alda-1 decreased ferrous iron and MDA contents and elevated GSH and SOD levels relative to the model group (Figures 5F–I, *P < 0.01*), consistent with the effects of Tan I that decreased ferrous iron and MDA contents and elevated GSH and SOD contents (Figures 5F–I, *P < 0.01*). Further analysis of CD31 expression suggested that Alda-1 promoted CD31 expression, consistent with the effect of Tan I that promoted CD31 expression (Figure 5J). Collectively, promotion of ALDH2 expression inhibits EC ferroptosis and enhances angiogenesis post-MI.

### 3.6 Tan I inhibits HUVEC ferroptosis and promotes angiogenesis by activating ALDH2 signaling

**FIGURE 6:**
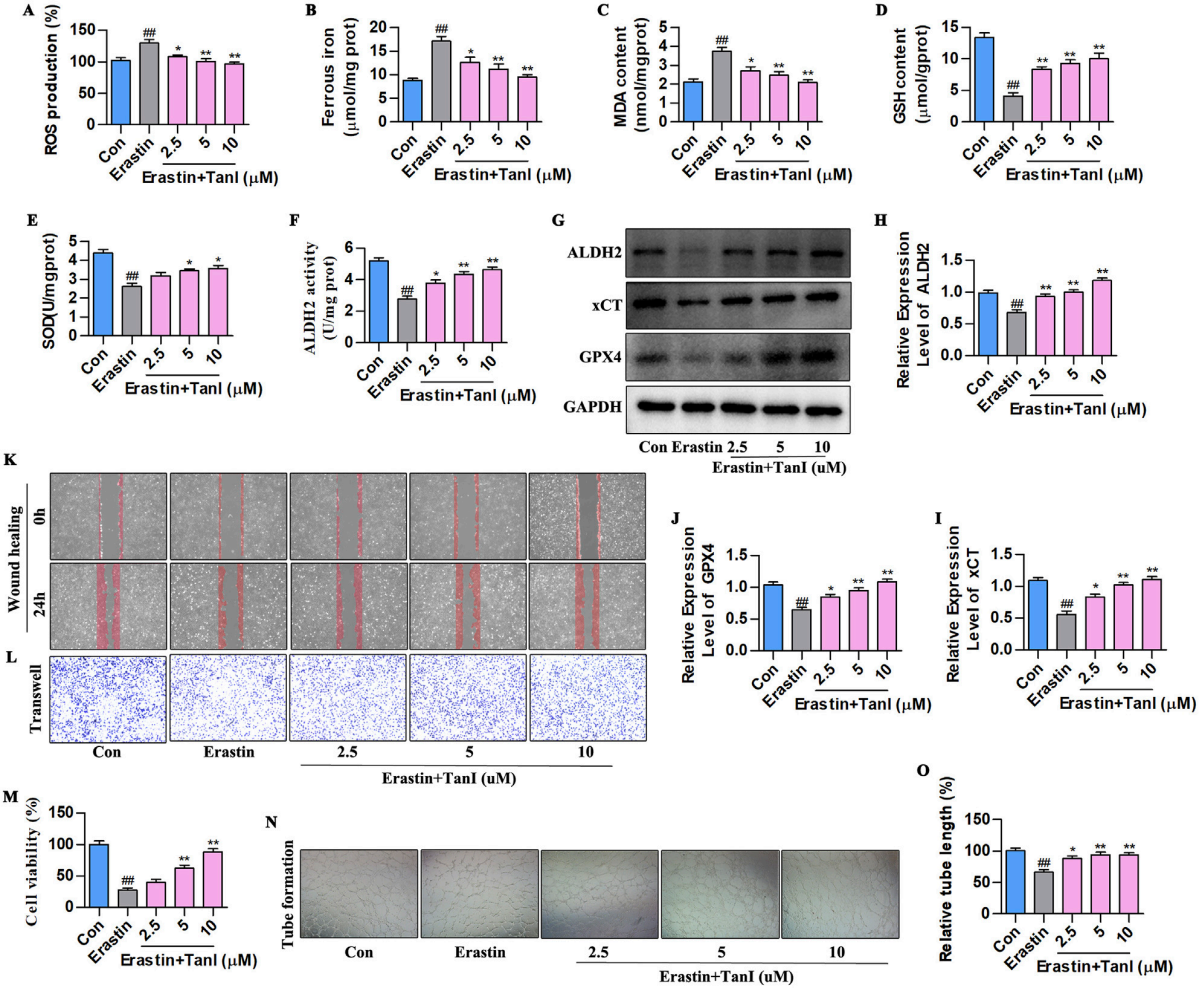
Tan I inhibits HUVEC ferroptosis and promotes angiogenesis by activating ALDH2 signaling. **(A–E)** ROS, Ferrous iron, MDA, GSH, and SOD contents (n = 3, *P < 0.01*). **(F)** ALDH2 activity (n = 3, *P < 0.01*). **(G–J)** ALDH2, xCT, and GPX4 protein levels were measured through western blotting (n = 3, *P < 0.01*). **(K,L)** HUVEC migration ability was determined using wound healing and Transwell assays. (M) CCK-8 assay (n = 3, *P < 0.01*). **(N)** Angiogenesis test. **(O)** Relative tube length (n = 3, *P < 0.01*). ^*#*^ *P < 0.05*, ^*##*^ *P < 0.01* vs. control group. *P < 0.05, **P < 0.01 vs. erastin group.

Also, erastin (10 µM) was utilized to construct a primary HUVEC ferroptosis model, and observe how ALDH2 signaling affected Tan I - mediated inhibition of HUVEC ferroptosis and promotion of angiogenesis. First, ferroptosis - related indicators were detected (Figures 6A–E). Ferrous iron, ROS, and MDA levels significantly elevated, while SOD and GSH levels markedly decreased compared with the control group (*P < 0.01*). Notably, consistent with *in vivo* results, Tan I reduced ferrous iron, ROS, and MDA contents, whereas elevated SOD and GSH levels dose-dependently (*P < 0.01*). ALDH2 activity and ALDH2 signaling-related proteins levels are shown in Figures 6F–J. Clearly, ALDH2 activity and ALDH2, xCT, and GPX4 protein levels markedly declined relative to the control group (*P < 0.01*), while Tan I markedly elevated their levels dose-dependently (*P < 0.01*).

Furthermore, we carried out CCK-8, wound healing, Transwell, and angiogenesis assays for detecting how Tan I affected angiogenesis. The CCK-8 assay showed that Tan I dose-dependently enhanced HUVEC proliferation (Figure 6M, *P < 0.01*). In the meantime, Transwell and wound healing assays demonstrated that Tan I dose-dependently promoted HUVEC migration (Figures 6K,L). As revealed by the angiogenesis assay, Tan I promoted tubular network formation in HUVECs (Figures 6N,O, *P < 0.01*). Consequently, Tan I inhibits ferroptosis and promotes angiogenesis by activating ALDH2 signaling.

### 3.7 Inhibiting ALDH2 enhances HUVEC ferroptosis to suppress angiogenesis

**FIGURE 7:**
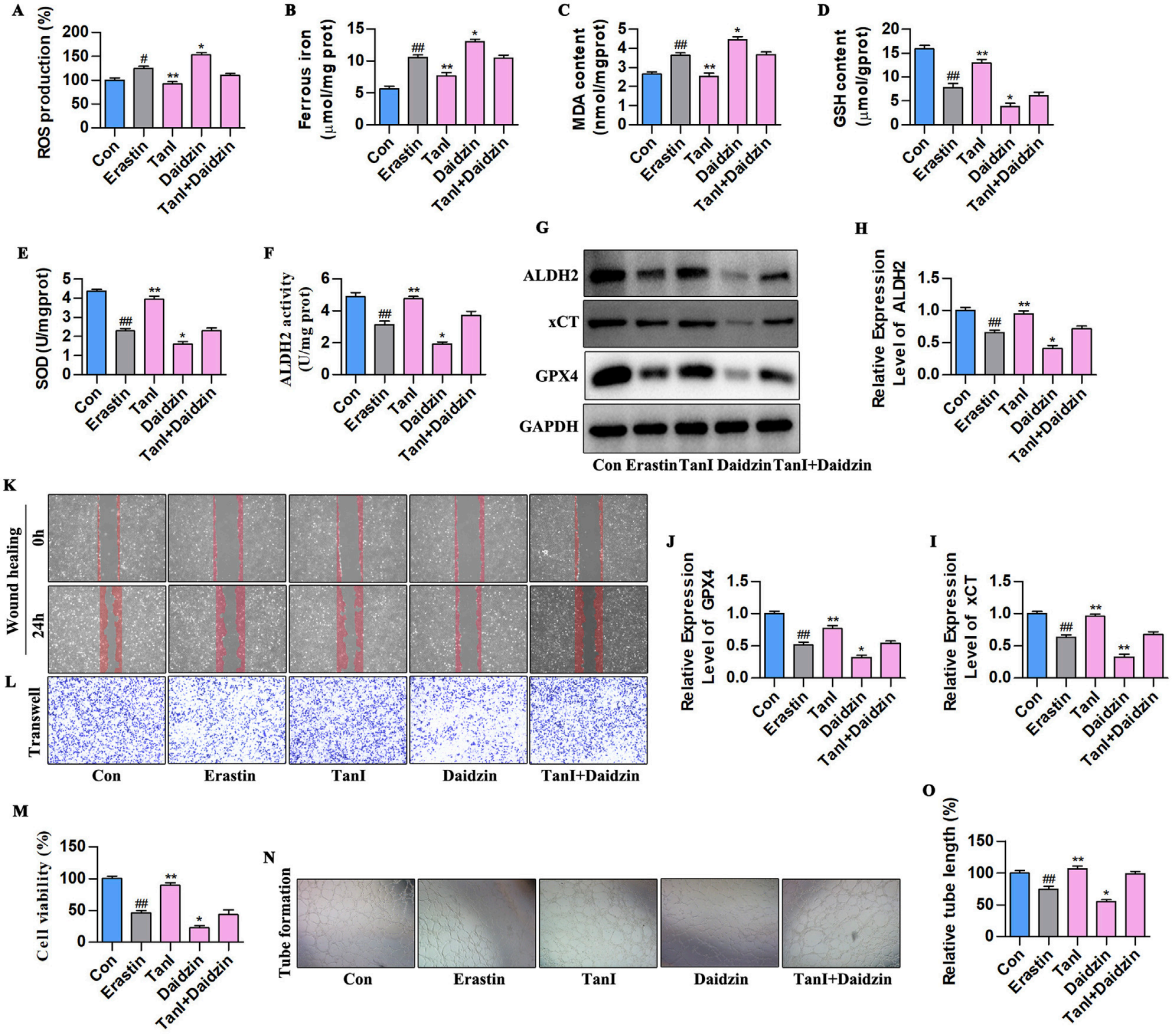
ALDH2 inhibition enhances HUVEC ferroptosis to suppress angiogenesis. **(A–E)** ROS, Ferrous iron, MDA, GSH, and SOD contents (n = 3, *P < 0.01*). **(F)** ALDH2 activity (n = 3, *P < 0.01*). **(G–J)** ALDH2, xCT, and GPX4 protein levels were detected through western blotting assay (n = 3, *P < 0.01*). **(K,L)** HUVEC migration ability was determined using wound healing and Transwell assays. **(M)** CCK-8 assay (n = 3, *P < 0.01*). **(N)** Angiogenesis test. **(O)** Relative tube length (n = 3, *P < 0.01*). ^*#*^ *P < 0.05*, ^*##*^ *P < 0.01* vs. control group. *P < 0.05, **P < 0.01 vs. erastin group.

We next verified how ALDH2 signaling affected Tan I-mediated HUVEC ferroptosis suppression and angiogenesis promotion. Ferroptosis - related indicators after daidzein intervention (10 µM) are displayed in Figures 7A–E. Obviously, ALDH2 inhibition increased ferrous iron, ROS, and MDA contents and decreased GSH and SOD levels (*P < 0.01*). Figures 7F–J present the ALDH2 activity and western blotting analysis results. It was obvious that ALDH2 inhibition suppressed ALDH2 activity and ALDH2 signaling-related protein levels (ALDH2, xCT, and GPX4) (*P < 0.01*). Additionally, angiogenesis-related indicators were analyzed, which showed that ALDH2 inhibition suppressed HUVEC proliferation (Figure 7M, *P < 0.01*), migration (Figures 7K,L), and tubular network formation (Figures 7N,O, *P < 0.01*). Taken together, these results reveal that ALDH2 inhibition promotes HUVEC ferroptosis to inhibit angiogenesis.

### 3.8 Promoting ALDH2 signaling inhibits HUVEC ferroptosis to enhance angiogenesis

**FIGURE 8:**
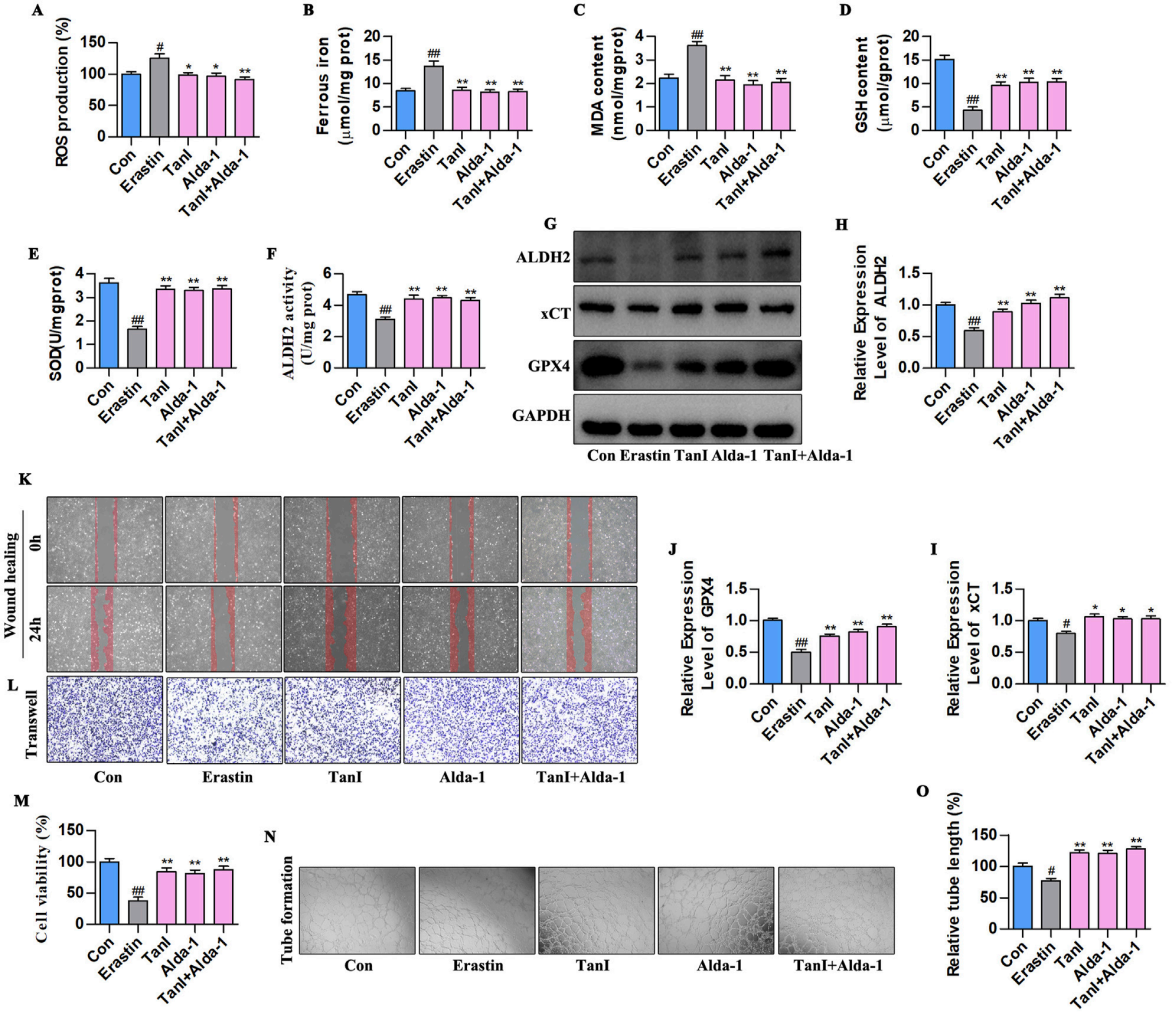
Promoting ALDH2 signaling inhibits HUVEC ferroptosis to enhance angiogenesis. **(A–E)** Ferrous iron, ROS, MDA, GSH, and SOD contents (n = 3, *P < 0.01*). **(F)** ALDH2 activity (n = 3, *P < 0.01*). **(G–J)** ALDH2, xCT, and GPX4 protein levels were detected through western blotting assay (n = 3, *P < 0.01*). **(K,L)** HUVEC migration ability was determined using wound healing and Transwell assays. **(M)** CCK-8 assay (n = 3, *P < 0.01*). **(N)** Angiogenesis test. **(O)** Relative tube length (n = 3, *P < 0.01*). ^*#*^ *P < 0.05*, ^*##*^ *P < 0.01* vs. control group. *P < 0.05, **P < 0.01 vs. erastin group.

Alda-1 was adopted for validating how ALDH2 signaling influenced the Tan I-mediated ferroptosis inhibition and angiogenesis promotion. According to Figures 8A–E, ALDH2 activation reduced ferrous iron, ROS, and MDA contents and elevated GSH and SOD levels (*P < 0.01*). The results of ALDH2 activity and western blotting assay in Figures 8F–J showed that ALDH2 activation enhanced ALDH2 activity and promoted the ALDH2 signaling-related protein levels (ALDH2, xCT, and GPX4) (*P < 0. 01*). In addition, based on the angiogenesis - related indicator measurements, ALDH2 activation promoted HUVEC proliferation (Figure 8M, *P < 0.01*), migration (Figures 8K,L), and tubular network formation (Figures 8N,O, *P < 0.01*). To sum up, promoting ALDH2 inhibits ferroptosis of HUVECs to enhance angiogenesis.

## 4 Discussion

MI remains a major global human health risk (Ganjehei et al., 2014). Despite the fact that reperfusion therapy reduces the MI area and improves ventricular function, the overall incidence of HF continues to increase, which is attributed to the adverse left ventricular remodeling (Ibanez and Rossello, 2019), while post-infarction revascularization or rapid opening of collateral circulation is the key to inhibiting this remodeling and improving post-infarction cardiac function (Wei et al., 2023). With societal development, the prevalence of MI shows an increasing trend, therefore, effective methods and therapeutic drugs that promote angiogenesis and prevent and treat ventricular remodeling post-MI are urgently needed. In recent years, Tan I has been extensively adopted for treating cardiovascular and cerebrovascular disorders because of its ability to activate blood circulation, remove blood stasis, and dilate coronary arteries (Guo et al., 2020). Furthermore, basic experiments have demonstrated that Tan I possesses cardiovascular protective effects, including anti-atherosclerosis, anti-inflammation, anti-oxidation (Wu Y. T. et al., 2024). According to our findings, Tan I inhibited HUVEC ferroptosis and promoted angiogenesis to improve ventricular remodeling post-MI. Therefore, based on Tan I’s pharmacological activities, a deep investigation into the mechanism by which it protects against EC damage has potential therapeutic implications for its application in treating CVDs.

In the pathological network of post-MI ventricular remodeling, EC ferroptosis characterized by iron-dependent lipid peroxide accumulation acts as a core link between cell death and impaired tissue repair (Zhang Z. et al., 2022; Lu et al., 2024). Previous studies indicated that iron-dependent lipid peroxidation specifically damages polyunsaturated fatty acids in endothelial membranes, suppressing eNOS activity, reducing VEGF secretion, and inducing VE-cadherin degradation to increase vascular permeability (Zhang H. et al., 2022; Zhu et al., 2024). This dysfunction diminishes new blood vessel density in the ischemic border zone and exacerbates myocardial energy failure via impaired vessel-cardiomyocyte paracrine signaling. Additionally, studies have demonstrated that EC ferroptosis is pivotal in coronary artery disease progression, particularly in MI, where ferroptosis inhibitors or iron chelators mitigate myocardial injury (Zheng et al., 2022). Given that severe cardiac dysfunction and injury typically emerge 4 weeks post-MI (Wu Y. T. et al., 2024), this timeframe was selected to assess the effects of Tan I. Our results demonstrated that Tan I activates ALDH2 signaling to bidirectionally regulate angiogenesis via the ALDH2/xCT/GPX4 axis: (i) enhancing xCT-mediated cysteine uptake and GPX4 expression to protect ECs from ferroptosis; (ii) upregulating CD31 and promoting vascular loop formation. The “endothelial protection-angiogenesis-myocardial repair” cascade highlights ferroptosis inhibition as both a critical node to block cardiomyocyte death and a core trigger for vascular repair in ischemic regions. This establishes an “angiogenesis-oriented” strategy to disrupt the “ischemia-remodeling” vicious cycle, with EC ferroptosis identified as a central mediator of post-infarction ventricular remodeling.

Molecular docking and MD simulations are widely used to predict targets of traditional Chinese medicine for disease treatment (Wang et al., 2022), and were used in this study to validate the Tan I-ALDH2 interaction. The complex binding energy was −8.36 kcal/mol, and Tan I remained stable at the center of ALDH2’s binding site until the end of the simulation, suggesting a strong binding capacity. In addition, Tan I formed hydrogen bonds with the GLN-349 residue of ALDH2, which is a critical component of the agonist-binding domain in ALDH2. Furthermore, MD simulations of the complex showed that the complex’s binding conformation was stable and that the molecular potential energy of the ALDH2-bound region of Tan I was higher than that of unbound Tan I. To further validate this interaction, SPR was employed to quantify the binding affinity in real time. SPR analysis revealed a dissociation constant (K_D_) of 1.91 × 10^−8^ M, indicating a high-affinity interaction between Tan I and ALDH2, which is consistent with the strong binding energy observed in molecular docking. These integrated results from molecular docking, MD simulations, and SPR indicate that Tan I directly targets ALDH2 as an agonist, providing a robust basis for subsequent functional experiments and mechanistic validation.

As a key mitochondrial aldehyde dehydrogenase, ALDH2 disrupts ferroptosis through multidimensional mechanisms, including (i) lipid peroxidation detoxification. ALDH2 catalyzes the conversion of toxic aldehydes (e.g., 4-HNE/MDA) into inactive carboxylic acids, directly interrupting lipid peroxidation-mediated membrane damage cascades. Additionally, it reduces substrate supply for ACSL4 (e.g., arachidonic acid), suppressing the initiation of phospholipid peroxidation at the source (Lamb et al., 2024). (ii) Antioxidant defense axis enhancement. By upregulating xCT to enhance cysteine uptake, ALDH2 promotes glutathione (GSH) synthesis and maintains GPX4 activity, forming an “ALDH2-GSH-GPX4” defense axis to synergistically scavenge ROS and disrupt the “iron-ROS-lipid peroxidation” positive feedback loop (Karunakaran et al., 2024). (iii) Mitochondrial homeostasis maintenance. ALDH2 preserves mitochondrial function by inhibiting mitochondrial permeability transition pore (mPTP) opening, thereby maintaining membrane potential and oxidative phosphorylation (Shen et al., 2024). Concurrently, it regulates iron transporters to reduce mitochondrial iron accumulation, weakening the foundation of Fenton reactions (Han and Zhai, 2025). Our *in vitro* and *in vivo* experiments confirm that ALDH2 activation inhibits endothelial cell ferroptosis and promotes angiogenesis by upregulating xCT/GPX4 expression, ultimately improving post-MI ventricular remodeling. These mechanisms highlight ALDH2 signaling as a critical hub linking ferroptosis inhibition and vascular repair. Targeting ALDH2 may simultaneously block oxidative damage and microcirculatory dysfunction, providing a novel therapeutic strategy for cardiovascular diseases and foundational evidence for optimizing post-MI ventricular remodeling treatments.

Fer-1 potently inhibits ferroptosis. In our previous studies, Fer-1 inhibited post-infarction cardiomyocyte ferroptosis and improved post-infarction ventricular remodeling (Wu et al., 2023). In this regard, this work used Fer-1 to verify ALDH2 signaling’s role in Tan I-mediated EC ferroptosis inhibition and angiogenesis promotion. Levels of ferroptosis-related indicators were consistent with expectations, showing that Fer-1 significantly suppressed EC ferroptosis, promoted angiogenesis and inhibited post-MI ventricular remodeling, comparable to the effects of Tan I. This study further confirmed that Fer-1 activated ALDH2 and upregulated the expression of ALDH2, xCT, and GPX4, which echoed the mechanism of Tan I. The present findings strongly suggest an association between MI and EC ferroptosis. Moreover, this process might involve the inhibition of ALDH2 signaling. Further, Tan I effectively suppressed EC ferroptosis, promoted angiogenesis, and improved ventricular remodeling post-MI by activating ALDH2 signaling.

While this study provides valuable insights into the cardioprotective effects of Tan I in post-MI ventricular remodeling by pharmacological techniques, including ALDH2 inhibitor daidzin, ALDH2 activator Alda-1, and ferroptosis-specific inhibitor Fer-1, several limitations warrant acknowledgment. First, the absence of ALDH2 knockout (ALDH2-KO) mouse models precludes direct confirmation of ALDH2 signaling’s essential role in mediating Tan I’s therapeutic effects. This gap limits definitive establishment of causality between ALDH2 activation and the observed improvements in angiogenesis and ventricular remodeling. Second, the long-term efficacy and safety profile of Tan I remains uncharacterized, as the study did not evaluate potential adverse effects or the durability of cardioprotection beyond the experimental timeframe. While our pharmacological strategies (daidzin inhibition/Alda-1 activation) strongly implicate ALDH2 in Tan I’s mechanism, the lack of genetic ALDH2 modulation tools (e.g., endothelial-specific ALDH2-KO models) primarily manifests in two critical shortcomings: inadequate target specificity with inherent off-target risks, and ambiguous tissue-specific mechanistic resolution, as systemic pharmacological administration cannot delineate ALDH2’s distinct roles in endothelial cells, cardiomyocytes, or other cell populations, thereby compromising mechanistic validation. Furthermore, unassessed long-term safety concerns, including potential ALDH2 overactivation-induced disruption of physiological aldehyde signaling require systematic evaluation. To address these limitations, we are currently developing endothelial cell-specific ALDH2-KO mice for rigorous validation in follow-up studies, coupled with comprehensive assessments of Tan I’s long-term safety. Additionally, this study has several limitations, including the reliance on animal models, the need for human data, the specificity and sensitivity of the ferroptosis markers used, and the absence of TEM analysis for mitochondrial ultrastructure and specific markers differentiating ferroptosis from apoptosis/necroptosis. Despite these constraints, this work significantly advances understanding of post-MI ventricular remodeling and Tan I’s therapeutic potential. By demonstrating Tan I’s capacity to activate ALDH2 signaling, suppress endothelial ferroptosis, and enhance angiogenesis, we identify a novel cardioprotective axis. These findings not only establish a theoretical foundation for Tan I’s clinical translation but also highlight that targeting ferroptosis through ALDH2 modulation represents a novel and promising therapeutic approach in post-MI treatment. Future investigations incorporating ALDH2-KO models and exploring complementary mechanisms will further solidify the translational relevance of these discoveries.

## 5 Conclusion

The present work observed and validated the impact of ALDH2 signaling on suppressing EC ferroptosis, promoting angiogenesis, and improving ventricular remodeling post-MI. The key findings are summarized below: (i) MI occurs with EC ferroptosis, (ii) post-infarction EC ferroptosis mediates ventricular remodeling, and its mechanism is probably associated with inhibiting ALDH2 signaling, and (iii) Tan I suppresses EC ferroptosis, promotes angiogenesis, and improves ventricular remodeling post-MI by activating ALDH2 signaling. This study reveals the unique advantages of Tan I in activating ALDH2 signaling, which not only broadens the application prospects of ALDH2 agonists, but also indicates that Tan I, as a potential ALDH2 activator, exerts a complementary effect on treating CVDs and provides new ideas and methods for future drug development.

## Data Availability

The original contributions presented in the study are included in the article/supplementary material, further inquiries can be directed to the corresponding authors.
